# Management of Esophago-Gastric Junction Carcinoma: A Narrative Multidisciplinary Review

**DOI:** 10.3390/cancers15092597

**Published:** 2023-05-03

**Authors:** Vincenzo Tondolo, Calogero Casà, Gianluca Rizzo, Mariavittoria Leone, Giuseppe Quero, Virginia Alfieri, Luca Boldrini, Milutin Bulajic, Domenico Corsi, Francesco Micciché

**Affiliations:** 1 U.O.C. di Chirurgia Digestiva e del Colon-Retto, Fatebenefratelli Isola Tiberina, Gemelli Isola, 00186 Rome, Italy; vincenzo.tondolo@fbf-isola.it (V.T.); virginiaalfieri@yahoo.it (V.A.); 2 U.O.C. di Radioterapia Oncologica, Fatebenefratelli Isola Tiberina, Gemelli Isola, 00186 Rome, Italy; calogero.casa@fbf-isola.it (C.C.); mariavittoria.leone@fbf-isola.it (M.L.); francesco.micciche@fbf-isola.it (F.M.); 3 U.O.C. di Chirurgia Digestiva, Fondazione Policlinico Universitario A. Gemelli IRCCS, 00168 Rome, Italy; giuseppe.quero@policlinicogemelli.it; 4 Università Campus Bio-Medico College, 00128 Rome, Italy; 5 U.O.C. di Radioterapia Oncologica, Fondazione Policlinico Universitario A. Gemelli IRCCS, 00168 Rome, Italy; luca.boldrini@policlinicogemelli.it; 6 U.O.C. di Endoscopia Digestiva, Fatebenefratelli Isola Tiberina, Gemelli Isola, 00186 Rome, Italy; milutin.bulajic@fbf-isola.it; 7 U.O.C. di Oncologia Medica, Fatebenefratelli Isola Tiberina, Gemelli Isola, 00186 Rome, Italy; domenico.corsi@fbf-isola.it

**Keywords:** esophago-gastric junction carcinoma, preoperative chemoradiotherapy, tailored treatment, mini-invasive surgery, multidisciplinary approach

## Abstract

**Simple Summary:**

The management of esophagogastric junction (EGJ) cancer is multidisciplinary, and due to its specificity, requires a high-volume center with high medical and surgical expertise. The aim of this narrative review was to critically analyze the evidence and guidelines in the literature and examine the tips and tricks in surgical and medical treatment to increase the long-term outcome of patients with this kind of tumor.

**Abstract:**

Esophagogastric junction (EGJ) carcinoma represents a specific site of disease, given the opportunities for multimodal clinical care and management and the possibilities of combined treatments. It encompasses various clinical subgroups of disease that are heterogeneous and deserve different treatments; therefore, the guidelines have progressively evolved over time, considering the evidence provided by clinical trials. The aim of this narrative review was to summarize the main evidence, which orientates the current guidelines, and to collect the main ongoing studies to address existing gray areas.

## 1. Introduction

Esophagogastric junction (EGJ) carcinoma represents an anatomical site of neoplasia that, increasingly and progressively, is being considered autonomously from other neighboring sites (such as cranially esophageal and caudally gastric neoplasms) in terms of perspectives for treatment and clinical management [1]. The global incidence of EGJ cancer has increased in recent years. For instance, in the United States, the number of patients with EGJ cancer has increased by four- to five-fold over the past two decades [2]. In fact, increases in the incidence of esophageal cancer, which in many registers include EGJ cancers, in white men were largely attributed to an increase of more than 400% in the incidence of esophageal adenocarcinoma between 1975–1979 and 2000–2004. A similar increase was also found for white women over the same period (men: 5.69 of 100,000 person-years, women: 0.74 of 100,000 person-years) [2]. EGJ cancer is the main site of this increased incidence in relation to the increased prevalence of pathological conditions such as gastroesophageal reflux, Barrett’s esophageal metaplasia, and a decline in Helicobacter Pylori prevalence.

From an anatomical and histopathological point of view, EGJ represents the boundary between the esophagus and stomach, i.e., the site where the esophageal squamous epithelium transits to a columnar epithelium of the cardiac stomach [3]. In Western countries, adenocarcinoma appears to be progressively increasing, while in Eastern Europe and Asia, squamous forms remain the most common esophageal and junctional neoplasia [4]. Tumors classifiable as EGJ cancers are adenocarcinomas, while squamous cell carcinoma originating from the EGJ are classifiable as distal esophagus squamous cell carcinoma and, therefore, follow the same clinical management as them.

EGJ cancer is defined as adenocarcinoma with an epicenter within 5 cm from the EGJ and can be classified according to the classification proposed by Sievert et al., which introduced a classification [5] according to the position of the lesion at the endoscopy relative to the EGJ. Siewert I tumors are lesions of the distal esophagus (often associated with Barrett’s esophagus), with their epicenter located 1 to 5 cm above the EGJ, and are generally treated in the same manner as esophageal squamous cell carcinoma. Siewert II tumors are limited to the cardiac region proper, are recognized as “true EGJ cancer,” and are located within 1 cm above and 2 cm below the EGJ. Siewert III tumors represent sub-cardiac cancer, are located between 2 and 5 cm below the EGJ, and are generally treated in the same manner as gastric cancer. Regarding staging, the 8th edition of the American Joint Committee on Cancer (AJCC) includes Sievert I and II tumors as afferent forms of the esophageal neoplasm classification, while Sievert III tumors are staged following stomach neoplasm classification. [6] In Japan, according to the Nishi classification, EGJ cancer is defined as a tumor with an epicenter located within 2 cm proximal or distal to the EGJ [7]. The latest edition of the UICC-TNM classification classifies a tumor with an epicenter ≤2 cm below the esophagogastric junction as esophageal cancer, while a tumor with an epicenter >2 cm below the junction is classified as gastric cancer.

The management of EGJ cancer is multimodal and radical surgery represents the mainstay for curative treatment of EGJ cancer. However, an optimal surgical approach remains controversial due to the complex lymphatic drainage pathway of cancer into the mediastinum and abdomen. Even after radical surgical resection, the prognosis of EGJ cancer remains worse than gastric cancer [8]. In fact, although data on the survival outcomes of patients with EGJ cancer are often obtained from studies that include patients with gastric or esophageal cancer, some studies have reported a five-year overall survival of approximately 50% for patients with locally advanced EGJ cancer [8].

The fundamental principle for adequate surgical treatment is an adequate anesthesiologic evaluation of the patient and accurate clinical–radiological staging of the neoplasm [9]. If pre-operative clinical staging is not sufficient, laparoscopy should be performed to identify occult metastases (frequent in Siewert’s type I and II cancers) or to perform peritoneal cytology that, if positive, in the absence of macroscopic peritoneal carcinomatosis, can represent a poor prognostic factor in EGJ cancers [10].

In the context of the surgical management of EGJ cancers, an adequate lymphadenectomy could be considered mandatory to guarantee the adequate anatomical–pathological staging of the neoplasm. A fundamental principle for an adequate lymphadenectomy is the accurate knowledge of lymph node stations in which the frequency of metastases is higher according to the site and classification of the EGJ tumor.

## 2. Lymphatic Flow of EGJ Cancer

### 2.1. Mediastinal Lymph Node

No clear evidence exists regarding the optimal extent of mediastinal lymph node dissection in EGJ cancer. In a study by Siewert et al. on type II or III EGJ cancer, the frequency of lymph node metastasis in the lower mediastinum was 15.5% [11]. In particular, as reported in the study of Kurokawa et al., the rate of mediastinal lymph node metastasis in type II EGJ cancer was 3.8% in the upper mediastinum, 7.0% in the middle mediastinum, and 11.4% in the lower mediastinum [12]. According to the Nishi classification, Kurokawa reported the rate of patients with metastasis in at least one of the lower, middle, and upper mediastinal nodes as 13.3%, 7.1%, and 6.1%, respectively [13]. In a more specific analysis of mediastinal lymph node involvement in EGJ according to the Nishi classification, the lower mediastinal lymph node, and in particular the N. 110 station (lower thoracic paraesophageal lymph nodes, 0.5–11.9%), had the highest frequency of lymphatic flow [14]. The rate of mediastinal lymph node metastasis varied with the length of esophageal involvement: a rate of lower mediastinal lymph nodes metastasis was reported to be 24.3% in esophageal involvement greater than 2 cm and 30.6% if esophageal involvement was greater than 3 cm.

### 2.2. Perigastric Lymph Node

Lymph node metastases from EGJ cancer are common in stations N. 1, 2, 3, 7, 8a, 9 and 11p; stations in the upper stomach (1, 2 and 3) have the highest rate of metastasis; nodes of the left gastric artery (N. 7) and supra-pancreatic nodes (N. 8a, 9 and 11p) have a relatively high rate of metastasis. Metastases in distal gastric nodes, such as 4d, 5 and 6, are rare, and the range of incidence is 0–2.7%, 0–1.7% and <5%, respectively [15]. However, when the tumor diameter is greater than 6 cm, the frequency of metastases in distal gastric nodes is 10.7% [13]. Therefore, the lymphadenectomy of distal gastric nodes should be not necessary for EGJ cancer smaller than 6 cm.

### 2.3. Para-aortic Lymph Node

Although they are generally considered for distant metastases, some studies analyzed the role of para-aortic dissection in EGJ cancer. In the study of Kurokawa et al., the rate of para-aortic (N. 16a2 station) lymph node metastasis was 4.7% but increased to 10.1% if the tumor diameter was greater than 6 cm [13]. JCOG9502 reported metastasis in the N. 16a2 station in 9.3% of cases in EGJ cancer with esophageal involvement <3 cm [16]. However, the performance of a para-aortic lymph node dissection did not seem to increase survival from EGJ cancer.

Based on the incidence of lymph node metastasis (Table 1), lymph node dissection in EGJ cancer should be a priority. The top priority for nodal dissection was for stations 1, 2 and 3, followed by stations 7 and 11p; subsequent priority was for stations 110, 16a, 9, 8a and 9. The dissection of nodes of the middle mediastinum (#107, #108) and periaortic stations (#16a2) depended on the tumor site and its dimension. According to the length of esophageal invasion, the JGCA Guidelines provisionally recommended a different lymph node dissection for EGJ cancer: upper gastric lymph nodes, supra-pancreatic lymph nodes, and lower mediastinal lymph nodes when the length of esophageal invasion was ≤4 cm. If the length of esophageal invasion was greater than 4 cm, middle and upper mediastinal lymph node dissection should be considered.

## 3. Principles of surgery of EGJ Cancer

### Surgical Management of EGJ Cancer Must Respect Some Principles

The radical macroscopic and microscopic dissection of cancer (R0 resection) with negative resection margins. This principle represents one of the most important prognostic factors for a better oncologic outcome: 5 y overall survival rates from 43% to 49% after R0 resection, from 0% to 11% after R1 (microscopic residue) resection, and from 0% to 4% in case of R2 (macroscopic residue) resection [11,17,18,19]. To obtain an R0 resection, several concerns should be considered regarding optimal esophageal resection margins. In fact, the length of the esophagus resected should be influenced by the anastomotic and reconstruction technique; moreover, the length in vivo before resection is different (50%) from the length after resection. A margin of at least 5 cm ex vivo is required, but, considering the risk of surgery and other factors that affect the prognosis, a proximal resection margin >2.0 cm on the resected specimen is recommended as an appropriate length and has been shown to be associated with the prognosis. In all cases, the intra-operative pathological examination of resection margins on the resection edge is mandatory and should be always performed [10].

Adequate lymph node dissection according to the Siewert type of EGJ cancer. Lymph node involvement represents the main prognostic factor in EGJ tumors, predicting the occurrence of local or distant recurrences. The role of lymphadenectomy is an adequate staging of cancer, reducing the recurrence and improvement in cancer-related survival [20]. The risk of lymph node metastases according to the tumor site and Siewert type of EGJ cancer should guide surgeons in the choice of a better surgical approach [21]. The number of harvested lymph nodes has been shown to be an independent predictor of survival after esophagectomy, and several analyses on large databases (SEER database (8), WECC database (9)) demonstrated that a greater extent of lymphadenectomy was associated with increased survival for all patients with node-positive cancers. According to this evidence, NCCN guidelines recommended the resection of at least 15 lymph nodes for patients with esophageal cancer after preoperative therapy [10].

## 4. Type of Surgical Procedures

Several experts recommend esophago-gastrectomy for Siewert I tumors and total gastrectomy for type III tumors. For type II tumors, the choice of a better surgical procedure has been debated; some advocate for esophago-gastrectomy, while others recommend an extended total gastrectomy with a transhiatal dissection of the posterior mediastinum.

### 4.1. Thoracoabdominal Esophagectomy (Ivor Lewis Esophagogastrectomy)

This approach consists of a combined abdominal approach (laparotomic or laparoscopic) and thoracic approach, with a right thoracotomy or thoracoscopy, with an intrathoracic esophagogastric anastomosis. The first step is represented by the mobilization of the stomach to create a conduit, preserving the right gastroepiploic artery, and by abdominal lymphadenectomy. During the intra-thoracic step, the esophagus is resected, and mediastinal lymph nodes are harvested. The final step is represented by the creation of an esophagogastric anastomosis above the azygos vein. The advantage of this approach is that a greater resection margin is ensured. Disadvantages of this technique are the intrathoracic location of the esophagogastric anastomosis, with a consequent high rate of morbidity and mortality if a leak occurs (higher than 65%) [22], and a high incidence of severe bile reflux (reported in 3–20% of patients) [23].

### 4.2. Thoracoabdominal Esophagectomy with Cervical Anastomosis (McKeown Esophagectomy or 3-hole Esophagectomy)

This procedure combines the transhiatal and transthoracic approach to optimize esophagectomy and thoracic lymphadenectomy, and it involves the creation of esophagogastric anastomosis in the neck and in the cervical region. The first step is the en-bloc resection of the esophagus and mediastinal and upper abdominal lymph nodes by a right postero-lateral thoracotomy (or thoracoscopy). Subsequently, the stomach is mobilized for use as a conduit by a laparotomy (or laparoscopy). The third step is the performance of esophagogastric anastomosis in the neck, at a higher level than Ivor Lewis, by a left cervical incision. The main advantage of this approach is the potentially easier management of a possible leak from esophagogastric anastomosis, which is in the neck. However, this potential advantage was not demonstrated by the evidence in the literature. In a recent meta-analysis comparing the Ivor Lewis (1857 patients) and McKeown (1434 patients) procedures, the Ivor Lewis procedure was associated with a lower rate of anastomosis leaks in all grades, a lower rate of recurrent laryngeal nerve injury and shorter length of hospital stay. Grade ≥2 anastomotic leaks, the chylothorax, postoperative mortality rate, operative time, blood loss, R0 resection rate, and lymph nodes examined were similar between the two groups [24]. Other potential advantages of McKeown esophagectomy are the lower incidence of reflux and the possibility of obtaining a larger proximal resection margin [25].

### 4.3. Transhiatal Esophagogastrectomy

This procedure is performed by an upper midline laparotomy incision and left neck incision. The thoracic esophagus is dissected through the diaphragmatic hiatus and the neck. Cervical esophagogastric anastomosis is created after a gastric pullup. Disadvantages of this approach include the inability to perform a full thoracic lymphadenectomy and the inability to visualize the midthoracic dissection [25].

### 4.4. Transhiatal Distal Esophagectomy with Total Gastrectomy

This procedure is performed by an abdominal approach; a total gastrectomy is performed with extended lymphadenectomy (perigastric, coeliac trunk, splenic artery, hepatic artery, and lower mediastinal nodes). The distal esophagus is resected via the diaphragmatic hiatus with access to the posterior mediastinum, and a Roux-en-Y reconstruction is generally made with esophago-jejunal anastomosis.

### 4.5. Transhiatal vs. Transthoracic Esophagectomy

Esophagectomy, with a transthoracic approach, has the advantage of a larger proximal resection margin and adequate mediastinal node dissection, but it is associated with a higher rate of surgical stress and higher rates of fatal complications compared to the transhiatal approach. On the contrary, the transhiatal approach represents a low-stress surgical procedure with a low risk of fatal complications but does not provide an accurate mediastinal node dissection. A randomized controlled trial was performed on 220 patients with distal esophageal and EGJ cancer to compare transhiatal esophagectomy, with lymph node dissection of the abdominal and mediastinal stations, to thoracoabdominal esophagectomy with both abdominal and mediastinal extended lymph node dissection. No differences were found regarding R0 resection rates, in-hospital mortality, and the rate of anastomotic leak. The transthoracic approach was better than the transhiatal approach regarding the number of lymph nodes harvested (31 vs. 16; *p* < 0.001) but was associated with a high rate of respiratory morbidity (57% vs. 27%; *p* < 0.001) [26]. No significant differences were found regarding the five-year disease-free survival and five-year overall survival [27]. In the JCOG9502 trial, comparing the transhiatal with the transthoracic approach, the transhiatal approach was associated with a better 5 y overall survival (52.3% vs. 37.9%), a lower morbidity rate (34% vs. 49%) and a lower rate of pneumonia (4% vs. 13%) [28]. A recent meta-analysis on nine retrospective studies and two RCTs involving 2331 Siewert type II EGJ cancer cases compared transhiatal and transthoracic surgical approaches [29]. Regarding the postoperative outcome, the transhiatal group experienced lower intraoperative blood loss, shorter hospital stays and a lower incidence of pulmonary complications than the transthoracic group; no differences were found regarding the duration of surgery, R0 resection rate, the number of dissected lymph nodes, perioperative mortality and morbidity rate, and an abdominal complication rate and anastomotic leak rate (4.4% in the transthoracic vs. 6.0% in the transhiatal group). Regarding the long-term oncologic outcome, the transhiatal approach seems to guarantee longer overall survival than the transthoracic approach both at three years and at five years, and this difference is higher for EGJ cancers with an esophagus invasion lower than 4 cm [29].

### 4.6. Total vs. Partial Gastrectomy

The necessity of a partial or total gastrectomy was investigated for Siewert II EGJ tumors. For Siewert type II tumors, the excision of the upper perigastric area, suprapancreatic nodes, and paraaortic nodes was necessary; the oncological necessity of lower perigastric nodes was relatively low, suggesting that total gastrectomy was unnecessary, and proximal gastrectomy or esophagectomy with gastric tube reconstruction was sufficient. Proximal gastrectomy with a transhiatal approach resecting the lower mediastinal nodes could be feasible from an oncological standpoint. In a recent meta-analysis, 12 studies (1734 patients) comparing total and partial gastrectomy in Siewert II/III EGJ cancer were analyzed [30]. Partial gastrectomy seemed to reduce the operative time and intraoperative bleeding and improve the long-term nutritional status. However, this procedure was associated with a risk of anastomotic stricture and reflux esophagitis. Moreover, the oncologic outcome was not different from the outcome after total gastrectomy. This metanalysis suggests performing a partial gastrectomy, which guarantees the same oncologic outcome as a total gastrectomy, preserving the nutritional status [30].

According to their metastatic lymphatic drainage pattern, Siewert type I tumors need to be treated by a thoraco-abdominal subtotal esophagectomy with proximal gastric resection. Siewert III cancers should be treated with total gastrectomy and D2 lymph node dissection, and the transhiatal resection of the distal esophagus. Siewert type II cancers can be treated both by an abdominal and transhiatal total gastrectomy with distal esophagectomy and esophagojejunostomy rather than by transthoracic subtotal esophagectomy (Ivor Lewis) with gastric tube reconstruction and high intrathoracic esophago-gastrostomy [31]. In the case of extensive gastric and esophageal infiltration by the tumor, a transthoracic subtotal esophagectomy, total gastrectomy, and reconstruction by colon interposition may be required. The crucial step during the abdomino-transhiatal approach in Siewert type II cancer is the esophageal margin status. A macroscopic tumor-free proximal resection margin of at least 2 cm should be obtained on the fresh specimen [32,33], and the anastomosis in the lower mediastinum need to be technically uncompromised in safe conditions. Some authors recommend performing, as a first step, the dissection of the distal esophagus and obtainment of a negative frozen section to enable the performance of a gastric pullup for transthoracic esophagectomy if the resection margin is involved [34]. If a correct resection margin cannot be obtained, transthoracic esophagectomy should be performed.

In surgery for EGJ, mini-invasive approaches (laparoscopic and robotic) seem to be associated with lower postoperative morbidity, quicker functional recovery, and better quality of life at 1 year from surgery. Regarding the oncological outcome, mini-invasive approaches seem at least non-inferior to the open approach [35,36,37,38]. The French trial MIRO, analyzing the role of the mini-invasive approach in the thoracic phase of esophago-gastrectomy, reported reduced postoperative morbidity and, in particular, respiratory complications [39]. A recent metanalysis analyzed the results of nine studies, making up a total of 2149 patients with EGJ cancer treated with laparoscopy (1136 cases) or open esophago-gastrectomy with a transhiatal approach (1013 cases) [40]. Compared with the open approach, the laparoscopic approach was associated with a longer operative time and less blood loss. No differences were found regarding the number of harvested lymph nodes. Laparoscopic surgery was associated with shorter postoperative hospital stays than the open approach, but no differences were found in terms of the overall rate of postoperative morbidity and mortality. No significant differences were found regarding the 2-year overall survival; moreover, the laparoscopic approach was associated with a better 5-year overall survival [40]. However, mini-invasive esophago-gastrectomy was considered an evolving treatment, and no randomized studies comparing laparoscopic and open approaches for transhiatal esophago-gastrectomy for EGJ cancer exist. Moreover, open esophagectomy may be preferred over a mini-invasive approach for certain patients with previous abdominal surgery, large and/or bulky tumors, a possibly unusable gastric conduit, and difficulty with lymph node dissection, and open procedures should be replaced with mini-invasive approaches in older patients or those with significant comorbidities [41,42,43].

## 5. The Role of Endoscopic Management

Early EGJ cancer, defined as a tumor invading the mucosa or submucosa regardless of lymph node involvement, represents 2–3% of all EGJ cancers and requires an accurate evaluation to precisely define the extension of the disease [44,45]. Several features of early EGJ cancer were analyzed and established as prognostic determinants for the risk of lymph node metastases. According to the depth of invasion (and the consequent risk of lymph node involvement), T1 cancers were divided into T1a (limited to the mucosa) and T1b (involving submucosa); moreover, according to the depth of mucosal infiltration, T1 was divided into M2 (invading the lamina propria) and M3 (invading into but not through the muscolaris mucosa), and T2 was divided into SM1 (penetrating the superficial one-third of the submucosa, <500μm), SM2 (penetrating into the intermediate one-third of the submucosa, 500–1000 μm), and SM3 (penetrating the deepest one-third of the submucosa, >1000 μm) according to the depth of submucosal infiltration [46]. In addition to the depth of submucosal invasion, other factors, such as poor differentiation and the presence of lymphovascular invasion (LVI), were analyzed as possible prognostic factors predictive of lymph node metastases [47,48,49].

According to NCCN guidelines, early disease (pTis, pT1a, selected superficial pT1b without lymph–vascular invasion) could be effectively treated with endoscopic resection (EMR or ESD) [50]. Endoscopic submucosal dissection (ESD) seems to be better than endoscopic mucosal resection (EMR) in terms of the en-bloc resection rate, rate of post-procedure major complications, and rate of local recurrences [51,52].

However, an accurate risk stratification, analyzing several cancer features, is mandatory for the endoscopic management of EGJ cancer. In a recent series by Nieuwenhuis et al., 120 endoscopic resections of early esophageal cancers were stratified into three categories of risk: high-risk intramucosal cancer (T1a, poor differentiation grade and/or LVI), low-risk submucosal cancer (T1b, sm1, good or moderate differentiation grade, no LVI) and high-risk submucosal cancer (T1b, sm2/3 and/or LVI). After 29 months of follow-up, the annual risk of metastases in the high-risk intramucosal cancer group was 6.9% and not significantly different from the other type of “early” cancer [53]. A recent risk stratification study evaluated the results and features of 248 resected submucosal (pT1b) esophageal cancers, creating an individual risk calculator for post-resection metastases (both lymph nodes and distant metastases). In this group of patients, the overall 5-year incidence of metastases was 30.9%, which increased with submucosal invasion depth, LVI, and larger tumors. Based on this evidence, the authors created a score to predict the risk; T1b sm1/2 tumors, smaller than 20 mm and without LVI, was the category with a lower risk of 5-year metastases, ranging between 5.9 and 7.3% [54].

## 6. Multidisciplinary Treatment of Locally Advanced EGJ Cancer

Surgery represents the cornerstone of radical treatment, and for early-stage disease (cT1 cN0 cM0), surgery alone is the treatment of choice. In the case of locally advanced EGJ cancer (cT2-cT4 or cN1-cN3, cM0), it is recommended by international guidelines that a neoadjuvant or perioperative treatment is administered [4,50,55,56,57].

### 6.1. Preoperative Chemoradiation

The advantages of neoadjuvant chemoradiation in operable disease was assessed by van Hagen et al. through the CROSS trial [58], which enrolled patients affected by resectable locally advanced esophageal, EGJ squamous cell and adenocarcinoma. Patients were randomized to receive surgery alone versus neoadjuvant chemoradiation therapy; it is noteworthy that about 25% of the recruited cohort was composed of patients affected by EGJ lesions. The study’s results showed better survival outcomes (3- and 5- years overall survival rates) with significant benefits in terms of prognostic elements at the histological examination (remarkable rate of pathologic complete response ((pCR, 29%)) for neoadjuvant treatment and an inferior incidence of pathological nodal involvement). The concomitant chemotherapy schedule was weekly carboplatin and paclitaxel, and the total dose of radiotherapy was 41.2 Gy in conventional fractionation. Chemoradiation treatment has been shown to be superior to neoadjuvant chemotherapy alone in the study conducted by Burmeister et al. [59], which randomized 75 patients in a phase II trial who received chemotherapy versus 35 Gy with chemoradiotherapy, achieving better histopathological outcomes (pCR rate and R1 resection) in the chemoradiation modality. Moreover, in a phase III trial, Stahl et al. [60] randomized 126 patients affected by adenocarcinoma of the lower esophagus and cardia (Siewert I-III) to receive chemotherapy versus chemotherapy and chemoradiation in a preoperative setting; the study showed a better pCR rate and a superior tumor-free lymph-node rate in the combined treatment arm.

### 6.2. Perioperative Chemotherapy

Perioperative chemotherapy is another approach that was proposed according to the results of the MAGIC trial [61]. In this study, Cunningham et al. randomized patients affected by gastric, EGJ, or distal esophageal cancer to receive three preoperative cycles and three postoperative cycles of chemotherapy (schedule: epirubicin, 50 mg per square meter of body surface area, day 1; cisplatin, 60 mg per square meter, day 1; and continuous infusion of fluorouracil, 200 mg per square meter per day for 21 days) versus surgery alone; the results of the trial showed benefits in terms of the overall and progression-free survival. This approach was proposed for EGJ adenocarcinoma, particularly for tumors classified as Siewert III [4,55].

### 6.3. Current Guidelines: The Choice of the Best Multimodal Approach

The progressive accumulation of evidence in support of neoadjuvant radiochemotherapy versus surgery alone from the CROSS trial [58] and perioperative chemotherapy versus surgery alone from the MAGIC trial [61] in different settings has led to the emergence of an important question that needs to be solved by international guidelines: which treatment approaches to propose to the patient. American guidelines recognize two different kinds of EGJ patients according to the Siewert classification: radiochemotherapy treatment is recommended for patients affected by Siewert I and II adenocarcinoma [50], while perioperative chemotherapy is recommended for patients with Siewert III EGJ adenocarcinoma and are treated inconsistently for the clinical management of gastric cancer [55]. European guidelines [4] propose both options for EGJ in the context of esophageal cancer, with the suggestion that, without unequivocal evidence, chemoradiation therapy should be proposed, as encouraged in several centers, in the case of patients with Siewert I and II, with perioperative chemotherapy recommended for patients affected by Siewert III EGJ cancer. In fact, according to the retrospective experience published by Anderegg et al. [62], patients affected by esophageal or EGJ cancer who underwent neoadjuvant chemoradiation had better compliance with an inferior frequency of severe toxicities compared to those who underwent perioperative chemotherapy, maintaining similar surgical (R0 surgery rate) and survival (median overall survival) outcomes; the authors did not stratify patients according to the Siewert classification, but analyzing the surgical approach, only one patient underwent total gastrectomy; therefore, a clear predominance of patients with Siewert I and II can be assumed. However, as this choice is not supported by high-level scientific evidence, randomized clinical trials are currently underway to assess and verify the validity of this approach.

### 6.4. Definitive Chemoradiation

Patients affected by non-metastatic locally advanced EGJ cancer without a surgical indication of the patient’s general conditions or with the unresectable disease should be candidates—if the clinical conditions allow—for definitive chemoradiotherapy. A total dose of 50–50.4 Gy should be delivered in conventional fractionation [50].

### 6.5. Adjuvant Multimodal Treatments

Definitive histopathologic examination after radical surgery should be discussed by a multidisciplinary team to evaluate the eventually occurring risk factors that may necessitate adjuvant treatment. If no pre-operative treatment is performed and the histopathologic findings show positive surgical margins, pT3 disease, positive nodes, patterns of lymphovascular/perineural invasion and high-grade disease, adjuvant chemoradiation treatment could allow for a lower risk of local recurrence [50,57,63]. NCCN guidelines [50] manage adjuvant treatments on the basis of the histology type, considering squamous histotype forms as neoplasms of the distal esophagus; therefore, we focused on adenocarcinomas of the EGJ. Adjuvant treatments of EGJ adenocarcinoma patients that did not undergo preoperative chemoradiation or perioperative chemotherapy should be proposed according to the following indications:

1. EGJ adenocarcinoma should be evaluated for adjuvant therapy in R0 resection if pathologically proved positive nodes are found or in the case of the pT3–pT4a stage. R0 pT2 pN0 EGJ adenocarcinoma should be evaluated for adjuvant chemoradiation only if high-risk factors are detected, such as poor differentiation, high-grade disease, lymphovascular/perineural invasion, or in the case of patients younger than 50 years.
2. R1 resected EGJ adenocarcinoma without any preoperative treatment should be evaluated for chemoradiation. In the case of R2 resection, chemoradiation or palliative management is recommended.

EGJ adenocarcinoma treated with perioperative chemotherapy or preoperative chemoradiation is recommended to:

1. Complete the three cycles of chemotherapy if received perioperatively after surgery in the case of negative margins.
2. Undergo re-resection or chemoradiation—if not previously performed—in the case of microscopic positive margins.
3. Undergo chemoradiation—if not previously performed—or best supportive care in the case of macroscopic positive margins.

In the adjuvant setting, American guidelines propose a total conventionally fractionated radiotherapy dose of 45–50.4 Gy [50].

## 7. Future Perspectives and Ongoing Studies

According to the complexity and anatomical specificity of EGJ cancer, several studies and trials are currently ongoing to define, validate, and further orientate the multidisciplinary tumor boards in the clinical management of this kind of patient; Table 2 summarizes the currently ongoing trials.

The main topic is represented by the management of locally advanced disease and, in particular, the choice of the preoperative approach compared and/or combined with the two available treatment options according to each clinical presentation:

1. A phase III study that compares, in patients affected by esophageal or EGJ adenocarcinoma, neoadjuvant chemoradiation with perioperative chemotherapy followed by surgery is the ESOPEC trial, and the primary endpoint is the overall survival [64]; this study evaluates, on the chemotherapy arm, four cycles of FLOT-schedule chemotherapy in preoperative settings and for cycles of the same chemotherapy after surgery versus neoadjuvant chemoradiotherapy according to the CROSS protocol [58].
2. Neo-AEGIS [65] is another randomized phase III trial that compares, in patients affected by adenocarcinoma of the esophagus or EGJ, perioperative radiotherapy according to the MAGIC scheme [61] with neoadjuvant chemoradiation according to the CROSS study [58]; the survival outcomes are the measured primary endpoint.
3. In the PREACT study, perioperative S-1- and oxaliplatin-based chemotherapy is compared to neoadjuvant chemoradiotherapy in EGJ adenocarcinoma and gastric cancer [66]; the primary endpoint is represented by the 3-year disease-free survival of patients.
4. The RACE study is a randomized phase III trial that studies progression-free survival in resectable patients affected by EGJ who are randomized to receive four cycles of preoperative FLOT chemotherapy followed by surgery and four cycles of postoperative chemotherapy versus two cycles of FLOT chemotherapy plus chemoradiation (with fluoropyrimidine and oxaliplatin concomitant to 45 Gy radiotherapy) followed by surgery and four cycles of postoperative FLOT chemotherapy [67].
5. The TOPGEAR trial compares, in patients affected by EGJ or gastric cancer, perioperative chemotherapy according to the MAGIC scheme (three preoperative and three postoperative cycles) with a multimodal approach based on the same scheme of perioperative chemotherapy plus fluoropyrimidine-based chemoradiation (two cycles plus chemoradiation in the preoperative phase, and three cycles in the postoperative phase) [68].
6. The PROTECT trial is a prospective randomized phase II study that evaluates different chemotherapy regimens (FOLFOX versus paclitaxel and carboplatin) as concomitant to the same radiotherapy schedule (41.4 Gy), measuring the short-term complete resection rate and safety in the neoadjuvant treatment of esophageal and EGJ (Siewert I-II) cancer [69].

Alongside improvements in the surgical technique, radiotherapy technology, and multidisciplinary strategy, the development of new types of drugs also offers interesting opportunities that need to be explored soon.

1. The use of nanotechnology as a chemotherapy carrier is a frontier of interest in which only phase-two trials are currently available [70,71].
2. As has been the case with many other malignancies, where the introduction of immunotherapy has changed the standard of care and patient prognosis, in the multimodal treatments of EGJ cancer, the evaluation of the role of immune checkpoints is in progress. The main evidence is directed toward assessing the role of immunotherapy in gastric and gastroesophageal cancer, cumulatively recruiting patients with gastric cancer and EGJ cancer [72]. Moreover, the KEYNOTE975 trial aims to evaluate the impact of pembrolizumab in combination with definitive FOLFOX or Cisplatin plus fluoropyrimidine chemoradiation to treat patients affected by esophageal or EGJ cancer [73].

In the era of precision medicine, in tandem with classifications based on the endoscopic location of the lesion, molecular characterization contributes to a better definition of EGJ cancer; however, currently, as far as the molecular profile is concerned, they show features similar to gastric adenocarcinoma with a particularly chromosomally unstable variant, with the difference being that esophageal and junctional adenocarcinomas often show disproportionate DNA hypermethylation [74]. According to the molecular classification, we can differentiate EGJ cancer into undifferentiated carcinoma (UC), chromosomal instability subtype (CIN), genomically stable subtype (GS), microsatellite instability subtype (MSI), Epstein–Barr virus (EBV) subtype [74]; but further studies are therefore ongoing, including proteomic analyses [75], to better characterize EGJ cancer and allow tailored therapeutic strategies with precision therapies.

**Table 2:** Summary of ongoing trials in EGJ cancer.

Name of the Trial	Phase	Site	Endpoint	Setting	Enrollment	Arm A	Arm B	Estimated Completation Data
**ESOPEC** [64]	III	EC ADC EGJ ADC (Sievert I-III)	OS	**NeoAdj**	438	**Neoadjuvant CRT** (CROSS) RT (41.4Gy/23fractions) and concurrent CT with Carboplatin and Paclitaxel (5 weeks).	**Perioperative CT** (FLOT) 5-Fluorouracil, Leucovorin, Oxaliplatin and Docetaxel. Repetition every 2 weeks (d15, q2w). 4 neoadjuvant cycles (8 weeks) prior to surgery and 4 adjuvant cycles (8 weeks) postoperatively are given.	June 2024
**Neo-AEGIS** [65]	III	EC ADC EGJ ADC	OS	**NeoAdj**	366	**Perioperative CT** (Modified MAGIC or FLOT) Modified MAGIC: 3 cycles of CT pre-surgery and 3 cycles post-surgery. Epirubicin, cisplatin or oxaliplatin and a choice of 5-fluorouracil or capecitabine. Each cycle lasts 21 days. FLOT: 8 cycles of CT in total, 4 cycles of CT pre-surgery and a further 4 cycles of CT post-surgery. Each cycle of CT lasts 14 days/2 weeks.	**Neoadjuvant CRT** (CROSS) RT (41.4Gy/23 fractions) and concurrent CT with Carboplatin and Paclitaxel (5 weeks) prior to surgery.	March 2023
**PREACT** [66]	III	GC ADCEGJ ADC (Sievert II-III)	DFS	**NeoAdj**	682	**Perioperative CT** (SOX) 3 cycles of neoadjuvant CT with S-1 and oxaliplatin Surgery 3 cycles of adjuvant CT with S-1 and oxaliplatin	**Neoadjuvant CRT** 1 cycles of S-1 + Concomitant S1 RT (45 Gy in 25 fr) + 1 cycles of S-1 Surgery 3 cycles of adjuvant CT with S-1 and oxaliplatin	December 2023
**PROTECT** [69]	II	EC (located under the carena, beyond 25 cm from the incisors) EGJ (Siewert I-II)	CRR and severe (grade ≥ 3) postop. morbidity/mortality	**NeoAdj**	106	**Neoadjuvant CRT** (FOLFOX) RT (41.4Gy/23 fractions) and concurrent every two weeks CT with Folfox scheme (5-Fluorouracil; Oxaliplatin and Folinic acid).	**Neoadjuvant CRT** (Carbo-Paclitaxel) RT (41.4Gy/23 fractions) and concurrent weekly CT with Carboplatin and Paclitaxel.	June 2023
**RACE** [67]	III	EGJ ADC	PFS	**NeoAdj**	340	**Perioperative CT** (FLOT) 4 cycles of neoadjuvant CT with FLOT	**Perioperative CT** **+** **Neoadjuvant CRT** 2 cycles of neoadjuvant FLOT. CRT consists of oxaliplatin 45 mg/m² weekly and continuous infusional 5-FU 225 mg/m² plus concurrent radiotherapy given in 5/week fractions with 1.8 Gy to a dose of 45 Gy over 5 weeks. 4 cycles of adjuvant FLOT	May 2028
**TOPGEAR** [68]	III	GC ADC EGJ ADC (Sievert II-III)	OS	**NeoAdj**	574	**Perioperative CT** (ECF) 3 Cycles of epirubicin, cisplatin and 5-FU Surgery 3 Cycles of epirubicin, cisplatin and 5-FU	**Perioperative CT** **+** **Neoadjuvant CRT** 2 cycles of neoadjuvant ECF. CRT consists of continuous infusional 5-FU 200 mg/m² (or Capecitabine 825 mg/m²) plus concurrent radiotherapy given in 5/week fractions with 1.8 Gy to a dose of 45 Gy over 5 weeks. 3 cycles of adjuvant ECF.	December 2026
**KEYNOTE O59** [73]	II–III	EC ADC EC SCC EGJ	OS EFS	**Def**	700	**Pembrolizumab** **+** **Definitive CRT** 8 cycles of Pembrolizumab 200 mg q3w + 5 cycles 400 mg q6w Def CRT FOLFOX of FP (Cisplatin + 5-FU) and 50 vs. 60 Gy in 25 vs. 30 fractions.	**Placebo** **+** **Definitive CRT** 8 cycles of Placebo q3w + 5 cycles q6w Def CRT FOLFOX of FP (Cisplatin + 5-FU) and 50 vs. 60 Gy in 25 vs. 30 fractions.	February 2027

## 8. Conclusions

The multidisciplinary management of EGJ cancer, by combining a surgical approach with chemotherapy and radiotherapy, is the established approach to tailor treatment to each clinical presentation of the disease. The optimization of treatments is currently in progress via the clarification of still-present grey areas through studies ate currently ongoing to investigate whether they are capable of completing the definition of how multimodal treatments can be integrated with each other. In this framework, new drug therapies and new radiotherapy techniques, in parallel with the development of new and innovative surgical approaches, can provide new perspectives for the clinical management of EGJ malignancies.

## Figures and Tables

**Table 1:** Incidence of abdominal and mediastinal lymph node metastasis in EGJ cancer (11–14).

Lymph Node Station	Median of Reported Incidence
*No. 1 (Right cardiac)*	40.9%
*No. 2 (Left cardiac)*	25.2%
*No. 3 (Lesser gastric curve)*	43.4%
*No. 4 (Greater gastric curve)* 4sa 4sb 4d	2.3% 2.2% 0.4%
*No. 5 (Supra-pyloric)*	1.2%
*No. 6 (Infra-pyloric)*	0.9%
*No. 7 (Left gastric artery)*	25.0%
*No. 8 (Common hepatic artery)*	4.9%
*No. 9 (Coeliac axis)*	10.9%
*No. 10 (Splenic hilus)*	4.7%
*No. 11 (Splenic artery)* 11p 11d	15.4% 2.9%
*No. 12 (hepato-duodenal ligament)*	0.7%
*No. 16 (Para-aortic)*	4.8%
*Abdominal hiatal field* No. 19 No. 20	4.9% 1.5%
*Upper mediastinal* No. 105 No. 106	0.5% 0%
*Middle mediastinal* No. 107 No. 108 No. 109	0.4% 2.0% 1.7%
*Lower mediastinal* No. 110 No. 111 No. 112	12.0% 3.7% 1.9%
